# Figla Favors Ovarian Differentiation by Antagonizing Spermatogenesis in a Teleosts, Nile Tilapia (*Oreochromis niloticus*)

**DOI:** 10.1371/journal.pone.0123900

**Published:** 2015-04-20

**Authors:** Yongxiu Qiu, Shaohua Sun, Tapas Charkraborty, Limin Wu, Lina Sun, Jing Wei, Yoshitaka Nagahama, Deshou Wang, Linyan Zhou

**Affiliations:** 1 Key Laboratory of Freshwater Fish Reproduction and Development (Ministry of Education), School of Life Science, Southwest University, Beibei, Chongqing, China; 2 South Ehime Fisheries Research Center, Ehime University, Funakoshi, Ainan, Ehime, Japan; Ecole Normale Superieure de Lyon, FRANCE

## Abstract

*Figla* (*f*actor *i*n the *g*erm *l*ine, *a*lpha), a female germ cell-specific transcription factor, had been shown to activate genetic hierarchies in oocytes. The ectopic expression of *Figla* was known to repress spermatogenesis-associated genes in male mice. However, the potential role of *Figla* in other vertebrates remains elusive. The present work was aimed to identify and characterize the functional relevance of *Figla* in the ovarian development of Nile tilapia (*Oreochromis niloticus*). Tissue distribution and ontogeny analysis revealed that tilapia *Figla* gene was dominantly expressed in the ovary from 30 days after hatching. Immunohistochemistry analysis also demonstrated that Figla was expressed in the cytoplasm of early primary oocytes. Intriguingly, over-expression of *Figla* in XY fish resulted in the disruption of spermatogenesis along with the depletion of meiotic spermatocytes and spermatids in testis. Dramatic decline of *sycp3* (synaptonemal complex protein 3) and *prm* (protamine) expression indicates that meiotic spermatocytes and mature sperm production are impaired. Even though Sertoli cell (*dmrt1*) and Leydig cell (*star* and *cyp17a1*) marker genes remained unaffected, *hsd3b1* expression and 11-KT production were enhanced in *Figla*-transgene testis. Taken together, our data suggest that fish *Figla* might play an essential role in the ovarian development by antagonizing spermatogenesis.

## Introduction

In mammals, primary sex determination is commenced by the presence or absence of the Y chromosome, which controls the fate of the bipotential gonad. [1, 2]. Numerous studies have shown that sex determining signaling molecules in gonadal somatic cells controls the primordial germ cell (PGC) differentiation and gonad formation. Various studies have also considerably promoted the contributions of germ cells in sex differentiation and gonad formation [3–5]. Loss of germ cells in the XX gonad preferably activates the postnatal transdifferentiation of granulosa cell lineage to Sertoli cells [6], which indicates the involvement of germ cells in suppressing the male pathway in females mice. *Figla* (*f*actor *i*n the *g*erm *l*ine, alpha), which encodes a germ cell-specific basic helix-loop-helix transcription factor, is also known to express more abundantly in female germ cells than male [7]. It was reported that mutation of *Figla* disrupts folliculogenesis and further imposes sterility in females [8, 9]. On the contrary, the ectopic expression of *Figla* in male germ cells has also resulted in the down-regulation of a subset of testis-associated genes, sterility with impaired meiosis and germ cell apoptosis. These aforementioned data demonstrate that *Figla* plays dual roles in both the activation of oocyte-associated genes and the repression of sperm-associated genes during normal postnatal oogenesis [10].

Teleosts are characterized by diverse sex determination mechanisms [11]. However, the majority of downstream sex-related factors in somatic cells of gonads remain conserved throughout the vertebrate kingdom. In teleosts, germ cells development in ovary undergo three main processes: (i) migration/patterning of PGCs, (ii) proliferation of germ cells, and (iii) meiotic initiation at the critical timing of sex determination [12]. Genes related to PGC migration and maintenance has been extensively investigated for their involvements in sex determination and differentiation in fish. Similar to mouse, depletion of PGCs in genetic female individual has also resulted in the development of sterile testis in zebrafish [13, 14], medaka [15] and tilapia [16]. Rodríguez-Marí et al. [17] reported that meiotic failure or excessive apoptosis of oocytes in adult zebrafish gonad impaired the fertility, suggesting that oocytes-derived signals are essential in promoting ovarian differentiation. Previous studies have detected robust expression of *Figla* in the early primary oocytes in several teleosts [18–20]. Moreover, in black porgy (*Acanthopagrus schlegeli*), *Figla* is even suspected of playing a critical role in controlling the transformation of Sertoli cell into follicle-like cells during the sex-reversal from male to female [20].

Tilapia, an economically important species for aquaculture with XX/XY sex determination system, is known to be an ideal model to study fish sex determination/differentiation due to the availability of monosex larvae, genome information and well-documented sexual development processes. Recently, several reports have demonstrated the essentiality of molecular signaling and estrogen production from somatic cells in tilapia sex determination/differentiation [21–23]. Meanwhile, the timing of meiotic initiation of germ cells in tilapia also demonstrates the evident sexual dimorphism, suggesting that meiotic germ cells might be involved in sex differentiation [24, 25]. However, the exact roles of meiotic germ cells in fish sex differentiation still remain elusive. To elucidate the potential role of meiotic oocytes in ovarian formation and maintenance, we characterized *Figla* and analyzed its expression profile at different stages of gonadal differentiation. Furthermore, over-expression of *Figla* in the XY fish was also carried out. Our data demonstrated that the ectopic expression of *Figla* in XY fish resulted in the depletion of spermatocytes and mature sperm, and down-regulated the spermatogenesis-associated gene expression. Our present work suggests that fish *Figla* might play an important role in ovarian development via repressing the expression of spermatogenesis-associated genes.

## Materials and Methods

### Fish

Tilapias were reared in large tanks with a re-circulating aerated freshwater system. Fish were maintained at ambient temperature (26°C) under natural photoperiod. All genetic females (XX) and males (XY) were obtained by artificial fertilization of eggs from normal female (XX) with sperms from either sex reversed male (XX) or super male (YY), respectively. The super males (YY) were obtained by crossing the normal XY-male with the XY-female, which was sex-reversed hormonally by E2 treatment. All experiments were conducted in accordance with the legal requirements of China. The Committee of Laboratory Animal Experimentation at Southwest University, China, approved all procedures and protocols related to treatment and maintenance of the animals.

### Identification of tilapia *Figla* gene

The deduced mRNA sequence including the open reading frame (ORF) and untranslated regions for *Figla* gene was isolated from the 3-month-old tilapia gonadal transcriptome database [26]. Gene specific primers were designed to amplify the mRNA of *Figla* gene from the ovary. All PCR products were ligated into the pGEM-T easy vector (Promega, USA) and sequenced at Life Technologies Corporation (Shanghai, China). Gene structure (exon-intron distribution) was deduced by aligning the mRNA sequence and tilapia genomic DNA sequence of *Figla* gene (retrieved from http://www.ensembl.org/Oreochromis_niloticus/Info/Index).

### Phylogenetic and syntenic analysis

A phylogenetic tree of Figla proteins was constructed using the tilapia Max (Myc-associated factor X) (XP_005475095.1) as an out-group. In brief, the deduced amino acid sequences of tilapia Figla and its homologous counterparts from other species were aligned using Clustal W. The neighbor-joining method was used to construct the phylogenetic tree using MEGA 5.0 [27]. The values represent bootstrap scores out of 1000 trials, indicating the credibility of each branch. The GenBank accession nos. of the sequences used in this study are as follows: tilapia (KP096546), tetraodon (*Tetraodon nigroviridis*) (ACH91670.1), medaka (*Oryzias latipes*) (NP_001098215.1), fugu (*Takifugu rubripes*) (NP_001177290.1), zebrafish (*Danio rerio*) (NP_944601.2), *Xenopus* (*Xenopus laevis*) (NP_001088667.1), lizard (*Anolis carolinensis*) (XP_008120436.1), human (*Homo sapiens*) (NP_001004311.2), lancelet (*Branchiostoma floridae*) (XP_002586647.1). The gene loci flanking *Figla* genes in the genomes of different vertebrates were determined using the ensemble genome browser (http://www.ensembl.org/index.html) with tilapia *Figla* (ENSONIG00000015856) as the query sequence. Then, their genomic location data was used to rebuild the synteny maps surrounding the *Figla* genes in vertebrates.

### Real-time PCR and statistical analysis

The primer sets used for real-time PCR were designed using Primer Express software (Applied Biosystems, USA) with at least one primer in each set flanking the intron-exon boundary, in order to prevent amplification from genomic DNA. Linear standard curves were generated with serial 10-fold dilutions with plasmids DNA containing the ORF of respective genes. All real-time PCRs were carried out in an ABI-7500 fast real-time PCR machine (Applied Biosystems, USA) following the manufacturer’s protocol. The PCR reactions were initiated by denaturation at 95°C (5 min), followed by 40 amplification cycles at 95°C (15 s) and 60°C (30 s). Dissociation protocols were used to measure the melting curves. The relative expression level (RNA abundance) was calculated by dividing the copy no. of target gene by the geometric mean of three reference genes (*eef1a1*, *actb* and *gapdh*). Data were expressed as means ± S.E.M. Significant differences (P<0.05) in the results were analyzed using Kruskal-Wallis ANOVA and GraphPad Prism 4 Software (GraphPad, USA).

### Tissue distribution and ontogenic analysis

For the tissue distribution analysis, three parallel samples from three adult fish were prepared to evaluate the expression of *Figla* gene. Briefly, total RNA was extracted from head kidney, kidney, brain, gill, heart, intestine, liver, ovary and testis of adult tilapia, according to the manufacturer’s instructions (TaKaRa, Japan). Total RNA (200 ng) from each tissue was reverse transcribed into first-strand cDNA using PrimeScript RT Master Mix Perfect Real Time Kit (TaKaRa, Japan) following the manufacturer's instructions. Real-time PCR was carried out according to the aforementioned method. Preliminary analysis showed no sexual dimorphic expression in tissues other than gonads, hence the non-gonadal tissues collected only from female fish were used in the tissue distribution analysis.

For ontogenic expression analysis, three independent gonadal samples of 5, 10, 20, 30, 40 and 50 days after hatching (dah) were used, and each sample was made from the gonadal pool of 50–100 fish. However, three individual fish gonads were used to prepare three separate samples of 60, 90, 120, 150 and 240 dah. All the procedures for RNA extraction, cDNA synthesis, real-time PCR and statistical analysis were carried out as described above.

### Western blotting

Western blotting was performed to analyze Figla expression in the gonads of four separate *Figla*-transgene XY fish and non-transgenic fish. Briefly, proteins were extracted from testes of both wild type XX/XY and *Figla*-transgene XY fish at 90 dah. Then, 150 ng of the whole gonad protien was separated by SDS-PAGE and transferred onto PVDF membrane (Amersham, Sweden). Then, the membranes were blocked with 5% low fat milk powder in TBST (20mM Tris-HCL pH7.5, 150 mM NaCl, 0.1% Tween 20) and incubated with primary antibody of Figla, and then with AP-labeled secondary antibody. Finally, the immunoreactive signals were stained with NBT/BCIP substrates and visualized on Fusion FX7 (Vilber Lourmat, France). Meanwhile, the expression level of Figla was normalized using β-Actin as reference protein.

To assess the specificity of antibodies including Figla, Dmrt1, Sycp3 and Cyp17a1 in tilapia gonads, Western blotting was carried out in both ovary and testis according to the same protocol. Characterization of StAR antibody in the ovary and testis had been reported previously [29].

### Immunohistochemistry (IHC)

The gonads were dissected from monosex (XX and XY) fish at 90 and 150 dah, *Figla*-transgene and control XY fish at 90 dah, then Bouin-fixed, paraffin-embedded, and cut into 5μm sections. These sections were deparaffinized, hydrated and processed according to the previous reports [28, 29]. The tissues sections were then treated in a blocking solution (Roche, Germany), incubated with hrGFP (Stratagene, Canada), Figla, Dmrt1, Sycp3, Cyp17a1 and StAR polyclonal antibody (1:1000) overnight at 4°C, respectively, and rinsed with 0.01 M PBS three times for 5 min per wash. Subsequently, the sections were incubated with a second antibody conjugated with horseradish peroxidase (Bio-Rad, USA) at 1:2000 for 30 min, washed with PBS and visualized using diaminobenzidine (Sigma, USA) as the substrate. Sections were counterstained with hematoxylin. Polyclonal antibodies used for IHC were produced by immunizing the New Zealand white rabbits with each of the purified recombinant proteins generated from the partial coding region of tilapia Dmrt1, Sycp3, Cyp17a1 and StAR genes. Sera were collected after immunizing the rabbits three times and purified by affinity chromatography on Sepharose 4BFast Flow resin coupled with the respective recombinant protein. In this study, all the images for IHC were acquired with a Zeiss Axio Imager Z2 microscope equipped with an AxioCam MRc digital camera.

### Over-expression of *Figla* in XY fish

The ORF and 3’-UTR of *Figla* was amplified from the ovarian cDNA of adult tilapia by using two gene specific primers with appropriate restriction sites. After double digestions by *Eco*RI and *Xho*I (New England Biolabs, USA), the PCR fragments were purified and ligated to the multiple cloning sites of pIRES-hrGFP-1a vector (Stratagene, Canada) digested by the same restriction enzymes. Subsequently, sequence analysis was carried out to confirm the orientation and accuracy of the insert. Finally, the constructs with correct insert were purified by using a QIAquick gel extraction kit (Qiagen, Germany), linearized by *Psh*AI and used for subsequent microinjection and transgene analysis.

Briefly, linearized plasmid DNA of *Figla*-pIRES1a-hrGFP was diluted in filtered 1XPBS solution to a final concentration of 80ng/μl, and injected into one-cell stage fertilized eggs under microscope using SYS-PV830 injector (WPI, USA). Subsequently, the genomic DNA was isolated from tail of each fish at 90 dah to check the genome integration. Furthermore, the fragment was sub-cloned into pGEM-T easy vector for sequencing analysis. The integration rate was calculated by dividing the number of individual carrying foreign DNA (*Figla*-pIRES1a-hrGFP) with total number of fish survived at 90dah. The abnormality was scored by calculating the ratio between the numbers of fish carrying transgene sequences and total number of transgenics with impaired spermatogenesis (depletion of meiotic spermatocytes and spermatids).

Finally, the injected XY fish were sacrificed at 90 dah in order to analyze the effects of *Figla* over-expression by GSI measurement, real-time PCR, histological observation by H.E. staining, Western blotting and IHC according to the protocols aforementioned. To trace the tissue specificity of *Figla* transgene, real-time PCR analysis was performed to compare the transcription level of *Figla* transgene in gonads and gonadectomised bodies of *Figla*-trasngene XY fish.

All the primer sequences used for molecular cloning and PCR amplifications were listed in S1 Table.

### Measurement of 11-KT levels by EIA

For EIA, a minimum of 150 μl of blood was drawn from the caudal vein of *Figla*-transgene and control XY fish at 90 dah. Blood samples were centrifuged at 10 000 g for 5 min at 4°C, and the supernatant was carefully collected. Finally, 11-KT levels in the serum were measured using the 11-KT EIA Kit according to the manufacturer’s instructions (Cayman, USA).

## Results

### Phylogenetic and syntenic analysis

**Fig 1:**
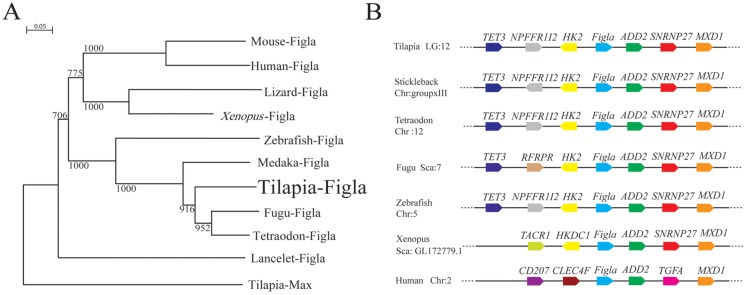
Phylogenetic and synteny analysis of *Figla* gene in vertebrates. (A) Phylogenetic tree representing the evolutionary relationship among vertebrate homologues of *Figla*, with tilapia Max (Myc-associated factor X) as an out-group. Values on the tree represent bootstrap scores from 1000 trials, indicating the credibility of each branch. Branch lengths are proportional to the number of amino acid changes on the branch. (B) Synteny maps showing the *Figla* locus organization in vertebrates. Gene symbols are described according to Ensembl database. The bar lengths are not proportional to the distances between genes. The direction of the arrow indicates the gene orientation.

Tilapia *Figla* cDNA was identified from the tilapia ovary, and its genomic sequence was obtained from tilapia genome website (http://www.ensembl.org/Oreochromis_niloticus/Info/Index). Comparative analysis of genomic DNA and mRNA revealed that tilapia *Figla* is located in LG12 and has 5 exons (S1 Fig). Multiple alignments demonstrate a well-conserved DNA binding domain of basic helix-loop-helix of Figla among all vertebrates (S2 Fig). Phylogenetic analysis shows that Figla from fish species cluster into one clade, while the amphibian, reptile and mammalian homologues formed a distinct clade (Fig 1A). Furthermore, synteny analyses reveal the organization of the genomic region surrounding *Figla* genes in tetrapods and teleosts. Comparative analysis of chromosomal organization shows that, *Figla* and its upstream gene, *HK2* (hexokinase 2), has a conserved synteny in teleosts and *Xenopus* (Fig 1B). In addition, conserved synteny of the downstream genes of *Figla* (*ADD2* (β-adducing) and *MXD1* (MAX dimerization protein 1)) was detected between human, *Xenopus* and teleosts (Fig 1B). In summary, *Fig1a* locus revealed a synteny of evolutionarily conserved genes in vertebrates, despite several local rearrangements and gene insertion in tetrapods.

### Tissue distribution

**Fig 2:**
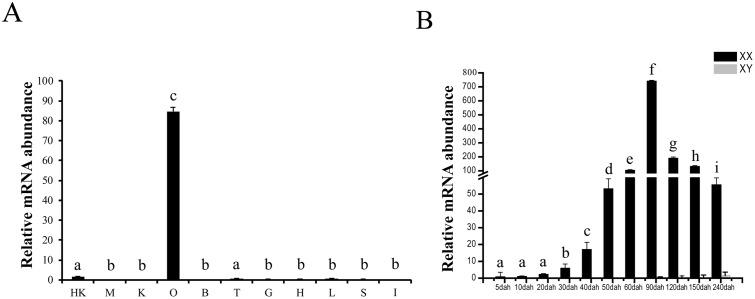
Expression profile of *Figla* gene in tilapia. (A) Expression of *Figla* in adult tilapia tissues by real-time PCR. HK, head kidney; M, muscle; K, kidney; O, ovary; B, brain; T, testis; G, gill; H, heart; L, liver; I, intestine; S, spleen. (B) Ontogenic expression of *Figla* gene in tilapia gonads analyzed by real-time PCR. Data were represented as means ± S.E.M. of three independent samples. The expression level was normalized using the geometric mean of the levels of three internal control genes (*gapdh*, *ef1a*, and *actb*). Different lower-case letters indicate a significant difference in *Figla* mRNA levels (P<0.05).

Real-time PCR was used to investigate the expression pattern of *Figla* gene in different tissues of adult tilapia. In consistence with mice [7], human [30], zebrafish [31] and medaka [32], tissue distribution analysis shows that *Figla* is dominantly expressed in the ovary, while it is barely detectable in other tissues in adult tilapia (Fig 2A).

### Expression of *Figla* gene during gonadal development

**Fig 3:**
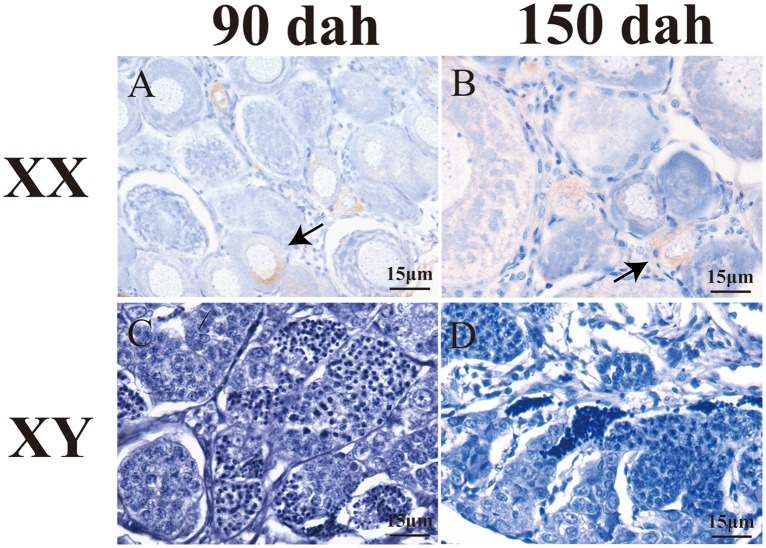
Cellular localization of Figla expression in tilapia gonads by IHC. Figla was abundantly observed in early primary oocytes at 90 (A) and 150 dah (B) of XX fish. No signal was detected in the control testis (C and D). Arrows indicate positive immunostaining.

Real-time PCR analysis revealed that mRNA abundance of tilapia *Figla* gene was remarkably increased from 30 dah, peaked at 90 dah, and decreased from 120 dah in the XX gonads. In contrast, transcript level of *Figla* gene remained undetectable during all stages in XY gonads (Fig 2B). Consistently, IHC experiments also demonstrate that Figla is expressed in the early primary oocytes at 90 and 150 dah in ovaries (Fig 3A and 3B). On the other hand, Figla specific signals could not be detected in testes at both stages (Fig 3C and 3D).

### Sequence confirmation and morphological changes in *Figla*-transgene XY tilapia

**Fig 4:**
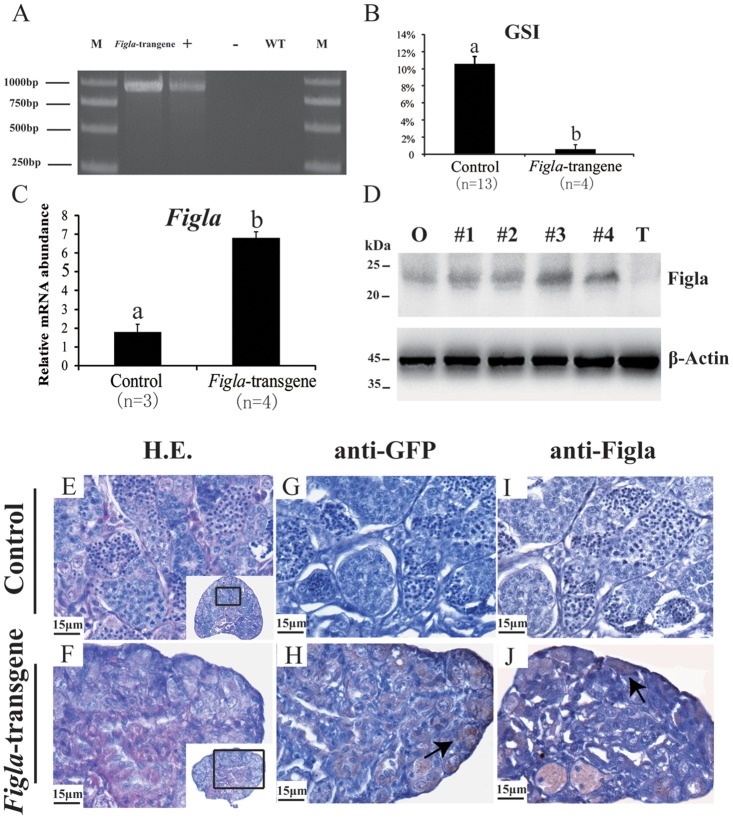
Screening, analysis and histological investigations of *Fig1a*-transgene fish. (A) Confirmation of transgene insertion by genomic PCR and sequencing. Lane 1 and 6, DNA marker; Lane 2, XY fish carrying *Figla*-transgene; Lane 3 and 4, using plasmid and water as template, respectively; Lane 5, XY control fish. (B) Comparison of GSI (Gonadal Somatic Index) of control and *Fig1a*-transgene XY fish. Data were expressed as means ± S.E.M. of control (n = 4) and *Fig1a*-transgene XY (n = 13) fish. Different letters indicate statistical differences at P<0.05. (C) Expression analysis of *Figla* gene in the testis of the *Fig1a*-transgene and control XY fish by real-time PCR. (D) Expression profiles of Figla in the gonad of control XX, *Figla*-transgene XY fish and control XY fish at 90 dah analyzed by Western blotting. β-Actin was used as reference protein to validate equal loading for Western blot analysis. O, Ovary; T, Testis; #1–4, four individuals of *Figla*-transgene XY. (E) Spermatogonia and all phases of spermatogenic cells could be detected in the testis of the control XY fish. However, only spermatogonia cells, but not later phase spermatogenic cells of spermatocytes and spermatids, were detected in *Fig1a*-transgene XY testes (F). Abundant expression of hrGFP (H) and Figla (J) were detected in the *Fig1a*-transgene XY testis, while no positive immunostaining was observed for hrGFP (G) and Figla (I) in the control testis. Arrows indicate positive immunostaining of respective genes. Inset represents the low magnified view of the respective gonads.

To clarify the potential functions of fish *Figla* in gonadal development and differentiation, the ORF and 3’-UTR of *Figla* gene were sub-cloned into the multiple cloning sites downstream of the CMV promoter in the pIRES-hrGFP-1a vector. After microinjecting into the one-cell stage fertilized egg, a specific band of 947 bp was amplified from the genomic DNA of injected XY fish by genomic PCR (Fig 4A). Sequencing results further confirmed that the amplified DNA fragment contains both pIRES-hrGFP-1a vector and *Fig1a* sequence (Data not shown). The integration and abnormality rate induced by over-expression of *Figla* were approximately 47.62% and 95%, respectively (S2 Table).

Morphologically, the *Figla*-transgene XY fish had shrunken gonads and significantly reduced GSI values, compared to the control XY fish (Fig 4B). Real-time PCR analysis revealed that expression of *Figla* gene is dramatically up-regulated in 3-month-old *Figla*-transgene XY fish compared to control group (Fig 4C). Moreover, real-time PCR results demonstrated a significant difference in *Fig1a* transcription between gonad and gonadectomised body of *Figla*-transgene XY fish, which indicated that over-expression of *Figla* gene was dominantly expressed in the gonad, but not other tissues (S3 Fig). Western blotting analysis revealed that a specific band around 22.5 kDa for Figla was detected from the total protein in the gonads of control XX and *Figla*-transgene XY fish, but it was barely detected in the testis of control XY fish (Fig 4D).

In tilapia, spermatogonia and all phases of spermatogenic cells could be detected in the testis of 3-month-old XY fish (Fig 4E). However, only spermatogonia cells, but not later phase spermatogenic cells, i.e. spermatocytes and spermatids, were detected in *Figla*-transgene XY testis (Fig 4F), indicating that over-expression of *Figla* impaired spermatogenesis. Moreover, strong expression of hrGFP (Fig 4H) and Figla (Fig 4J) were detected mainly in the areas of spermatogonial cells of *Figla*-transgene XY fish, but not in control XY (Fig 4G and 4I), which suggested the efficacy and specificity of *Fig1a* over-expression.

### Molecular variations in *Figla*-transgene XY tilapia

**Fig 5:**
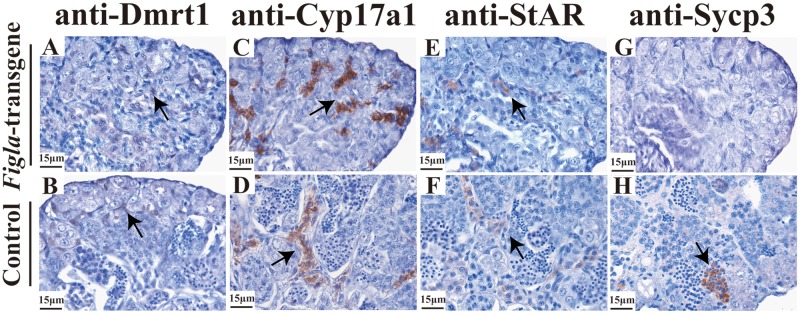
Effects of *Fig1a* over-expression on subcellular re-organization of somatic and germ cell markers. No significant difference of Dmrt1, Cyp17a1 and StAR were detected between *Fig1a*-transgene (A, C, E) and control group (B, D, F). Abundant expression of Sycp3 was detected in the testis of the control fish (H), while no positive immunostaining for Sycp3 was detected in the testis for the *Figla*-transgene fish (G). Arrows indicate positive immunostaining for each gene.

**Fig 6:**
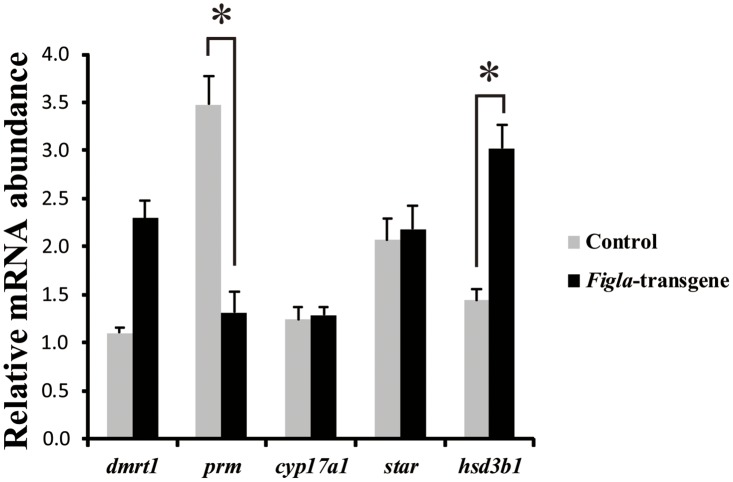
Comparative transcriptional profiling of *Figla*-transgene and control XY fish. Expression of *dmrt1*, *prm*, *cyp17a1*, *hsd3b1* and *star* in *Figla-*transgene and control fish at 90 dah were measured by real-time PCR. Relative mRNA levels were represented as means ± S.E.M. of 3 independent samples for the control fish and 5 samples for the *Figla*-transgene fish. *, indicates the significant difference (P<0.05) between the control and *Figla*-transgene fish.

Consistent with the histological observations, IHC also shows the absence of Sycp3 expression (Fig 5G) compared to the control group (Fig 5H). However, over-expression of *Figla* in XY fish didn’t alter the expression of *Dmrt1* (*Doublesex-* and *Mab-3*-related transcription factor-1) in the Sertoli cells in comparison with control XY fish (Fig 5A and 5B). Meanwhile, no significant difference of both *Cyp17a1* (cytochrome P450, family 17, subfamily A, type 1) and *StAR* (Steroidogenic acute regulatory protein) are detected in the Leydig cells between *Figla*-transgene (Fig 5C–5E) and control (Fig 5D–5F) groups. The IHC data are further supported by unaltered *dmrt1*, *star* and *cyp17a1* expression by real-time PCR analysis between the *Figla-*transgene and control fish (Fig 6). More interestingly, the expression of *prm* (protamine), which is specifically found in mature sperm, is significantly down-regulated in the *Figla*-transgene fish (Fig 6). The specificity in the gonads of Cyp17a1, Dmrt1, Figla, Sycp3 antibodies used for IHC was analyzed by Western blotting analysis. The results demonstrated that each antibody recognized a unique band in ovary and/or testis (S4 Fig).

### 11-KT level affected by *Figla* over-expression

**Fig 7:**
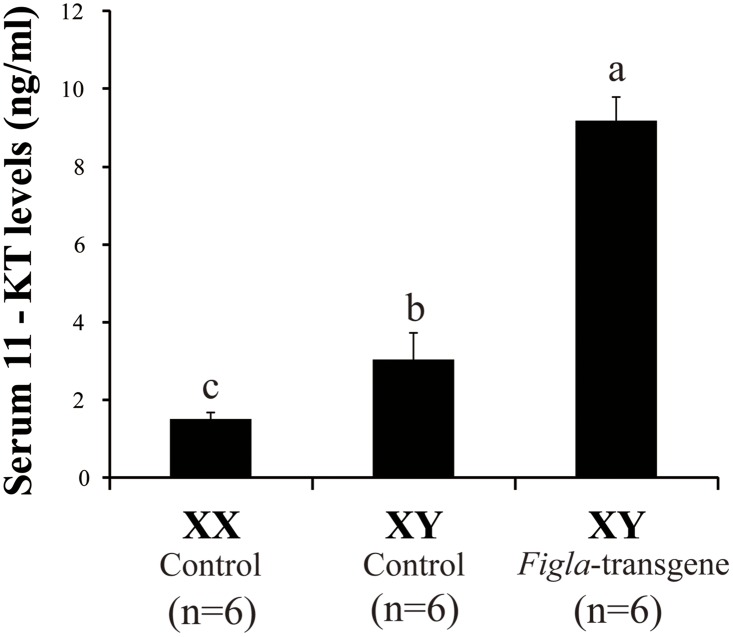
Comparison of serum 11-KT levels between *Figla*-transgene and control XY fish. Results were presented as means ± S.E.M. of 6 independent samples, respectively.

11-KT is the major androgen found in the serum of tilapia. To determine whether *Figla*-transgene could affect androgen production or not, we collected blood samples from both *Figla*-transgene and control XY fish at 90 dah, and measured the 11-KT levels using EIA. Surprisingly, serum 11-KT levels are significantly elevated in the *Figla-*transgene XY fish (Fig 7).

## Discussion

In this study, we showed that *Figla* was a female specific gene expressed in the early primary oocytes. The synteny analysis depicted a relatively conserved locus organization surrounding *Fig1a* gene throughout vertebrates. Surprisingly, over-expression of *Figla* in XY fish led to a defect in spermatogenesis. Hence, we provide evidences that *Figla* is an effective antagonistic factor which represses spermtogenesis, and in turn contributes to ovarian development.

In mammals, sex determination and differentiation is determined by the antagonistic and balancing actions between male and female genes [10, 33]. Meanwhile, coordination between follicles and oocytes are indispensable for ovarian differentiation [34]. *Figla* encodes a germ cell-specific bHLH transcription factor that was initially identified by its ability to co-ordinate the expression of the oocyte-specific zona pellucida genes and folliculogenesis [7]. The tilapia Fig1a possesses a conserved bHLH domain, which suggestes the similar regulatory mechanisms in fish. Moreover, both phylogenetic and conserved syntenic analysis further indicate tilapia *Figla* is the homologous gene of its mammalian counterparts, suggesting its conserved role in fish ovarian differentiation.

Previous studies demonstrated that Figla was essential for folliculogenesis in mouse [7], human [35]. Likewise, in our study, tissue distribution analysis also revealed that tilapia *Figla* gene was dominantly expressed in ovaries, indicating its vital roles in fish ovarian differentiation. In mice, the expression of *Figla* was first detected at embryonic stage 13, peaked two days postpartum and decreased markedly by 7 and 14 days after birth, while it was barely detectable in adult ovaries [8]. In zebrafish, two peaks of *Figla* gene were found at 18 day post hatching (dph) and 30 dph [18]. Consistently, we also found significant up-regulation of *Figla* gene in ovaries around 30 dah (just after the onset of sexual dimorphism), which peaked at 90 dah (the critical period of oogenesis) and declined from 120 dah, which suggests the essentiality of *Figla* gene in both folliculogenesis and oogenesis in tilapia.

Several reports revealed that Rspo1 and Foxl2 activated pathways in somatic cells are indispensable in promoting and ensuring ovarian differentiation in vertebrates [36, 37]. Meanwhile, cross-talk between oocyte and ovarian follicular is of vital importance for fertility [34]. Previous investigations indicated that germ cells are definitely required to represses the male pathway and promote ovarian differentiation [38]. In mice, loss of germ cells in the XX gonad triggered the postnatal trans-differentiation of granulosa cell to Sertoli cells and promoted the development of testicular cord-like structures [15, 39]. In fish, disruption of PGC population by gene knockdown or chemical treatment resulted in trans-differentiation from pre-follicles into Sertoli and Leydig cells, and further masculinization of female gonad, which highlighted the importance of germ cells in female fertility [13, 15, 25, 40]. Therefore, the interaction between germ cells and somatic cells might also be critical for female sex differentiation in fish [13]. *Figla*, a key factor in the genetic hierarchies of germ cells, was shown to be an activator of oocytes genes during postnatal oogenesis in mammalian species [7]. Mice lacking FIGLA have normal embryonic gonad development, but without primordial follicles and loss of germ cells within days of birth, which resulted in massive depletion of oocytes, shrunken-ovaries and sterility [8]. Ectopic over-expression of *Figla* in male germ cells in transgenic mice led to defective spermatogenesis and down-regulation of a set of testis-associated genes [10]. Similar to *Fig1a* over-expressed male mice, our histological data also showed severely disrupted spermatogenesis in male tilapia at 90 dah. In contrast to normal 3-month-old XY fish, the reduced GSI index suggested that over-expression of *Figla* severely impaired gonad differentiation and development. Noticeable morphological defects further supported that over-expression of *Figla* arrested the differentiation of spermatocytes and sperm maturation. The defective meiotic progression was further confirmed by the reduced Sycp3 expression. Meanwhile, the reduced *prm* levels in *Fig1a*-transgene XY fish were also suggestive of similar phenomenon. In addition, knockout of *Figla* by TALENs (Transcription activator-like effector nucleases) resulted in gonadal masculinization with the down-regulation of female specific genes and up-regulation of male specific genes (data not shown). Taken together, we hypothesized that *Figla* might play essential roles in ovarian differentiation and contribute towards female development by both activating the female specific genes and antagonizing the spermatogenesis associated genes.

Previous reports in mice have shown that *Figla*-transgene disrupted spermatogenesis only from 5 months after birth [10]. In contrast to this report, defective spermatogenesis was observed in *Fig1a* over-expressed XY fish from 90 dah, when the meiotic initiation just occurred, suggesting a differential regulatory mechanism. In *Figla*-transgene male mice, the property of somatic cells and the expression of Sox9 in Sertoli cells were normal in the testes [10]. Likewise, no significant difference of *dmrt1* gene expression was noticed, indicating that over-expression of *Figla* didn’t impair the properties of Sertoli cells. In testis, Leydig cells are the major resources for steroidogenesis in fish [41]. Contradictory to the results in mice, we observed enhanced *hsd3b1* expression and 11-KT circulation in *Fig1a* over-expressed XY tilapia, even though the expression of *cyp17a1* and *StAR* in *Figla*-transgene XY fish in the Leydig cells remained constant. We speculate that excess 11-KT production impaired spermatogenesis, as high levels of androgen led to early puberty, shrinkage of testes, and in some cases, infertility in males [42]. Meanwhile, the increased 11-KT level in these fish could also be a compensatory response due to the inhibition of spermatogenesis. Taken together, our data suggests that *Fig1a* arrests the male gonadal differentiation, possibly by manipulating the steroid production and/or meiotic regulation.

In summary, our particular investigations showed the female specificity of Figla in the early primary oocytes. Over-expression of female specific *Figla* gene in XY fish eventually arrested spermatogenesis and sperm production. Therefore, fish *Figla* probably might be an antagonist of genetic hierarchies of male, which in turn favors ovarian development. However, further in-depth investigations are required to verify the regulatory mechanisms.

## Supporting Information

S1 Fig Schematic representation of the gene structure of tilapia *Figla*. (TIFF)

S2 Fig Multiple alignments of the deduced amino acid sequences of tilapia Figla with its counterparts in other vertebrates. (TIFF)

S3 Fig Comparison of *Figla* gene expression in gonad and gonadectomised body in *Figla*-transgene XY fish by real-time PCR. (TIFF)

S4 Fig Specificity of Figla (A), Cyp17a1 (B), Dmrt1 (C), Sycp3 (D) antibody in the gonads analyzed by Western blotting. (TIFF)

S1 Table Primer sequences used for molecular cloning and PCR amplification. (DOC)

S2 Table Integration and abnormality rates of *Figla*-transgene XY tilapia. (DOC)
