# Supplementary figures and images for: Figla Favors Ovarian Differentiation by Antagonizing Spermatogenesis in a Teleosts, Nile Tilapia (Oreochromis niloticus)
Source: PLoS One. 2015 Apr 20;10(4):e0123900. doi: 10.1371/journal.pone.0123900 (PMC4404364; doi:10.1371/journal.pone.0123900)

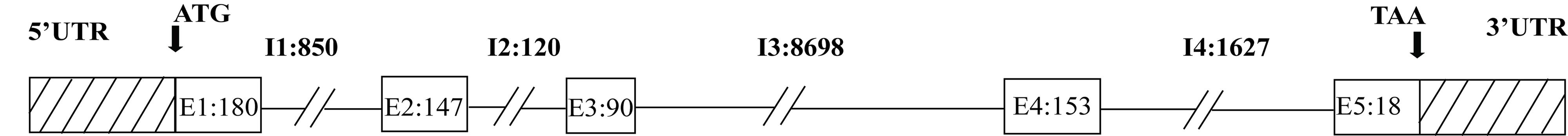

Supplement: S1 Fig — (TIFF) [file pone.0123900.s001.tiff]

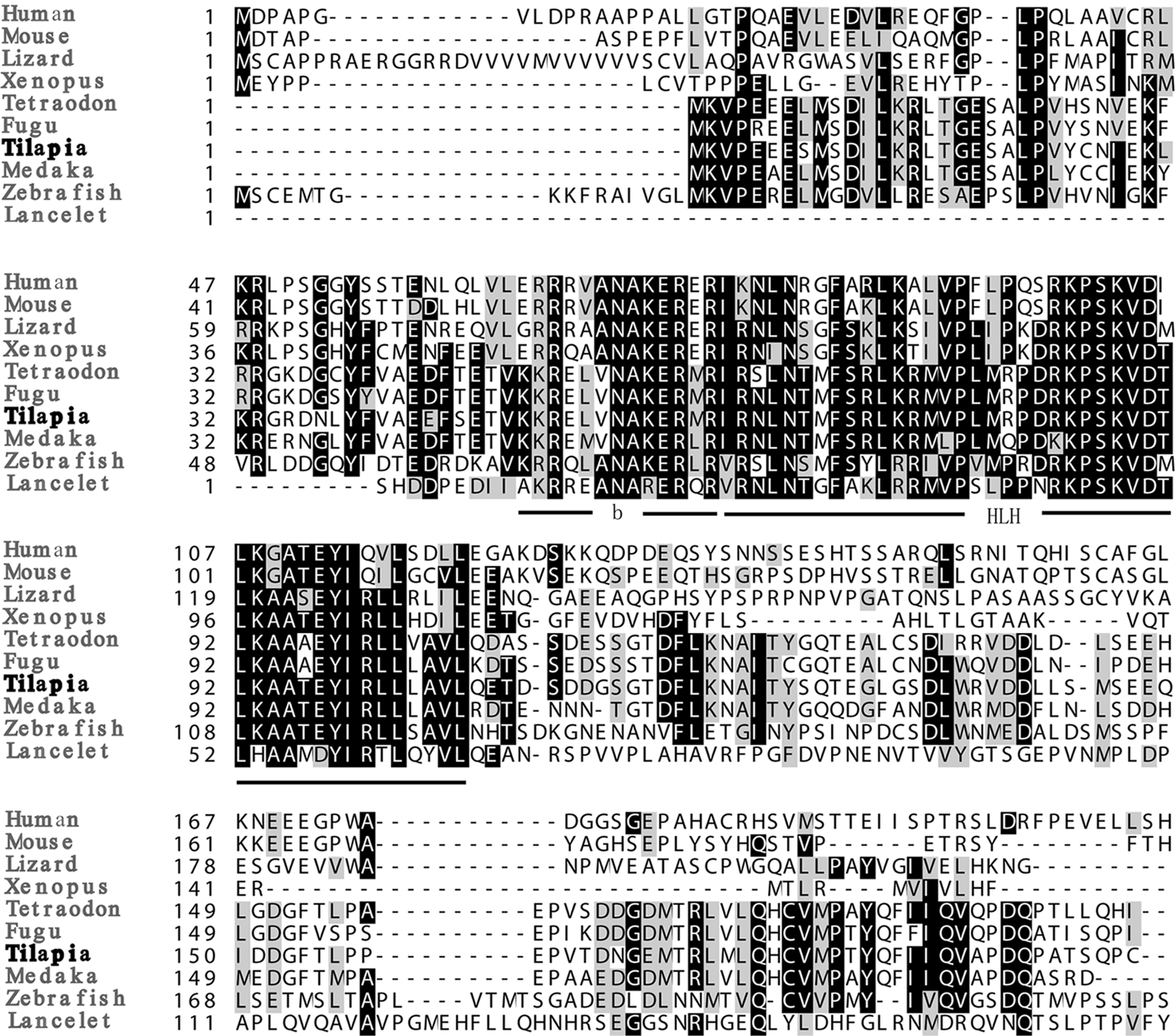

Supplement: S2 Fig — (TIFF) [file pone.0123900.s002.tiff]

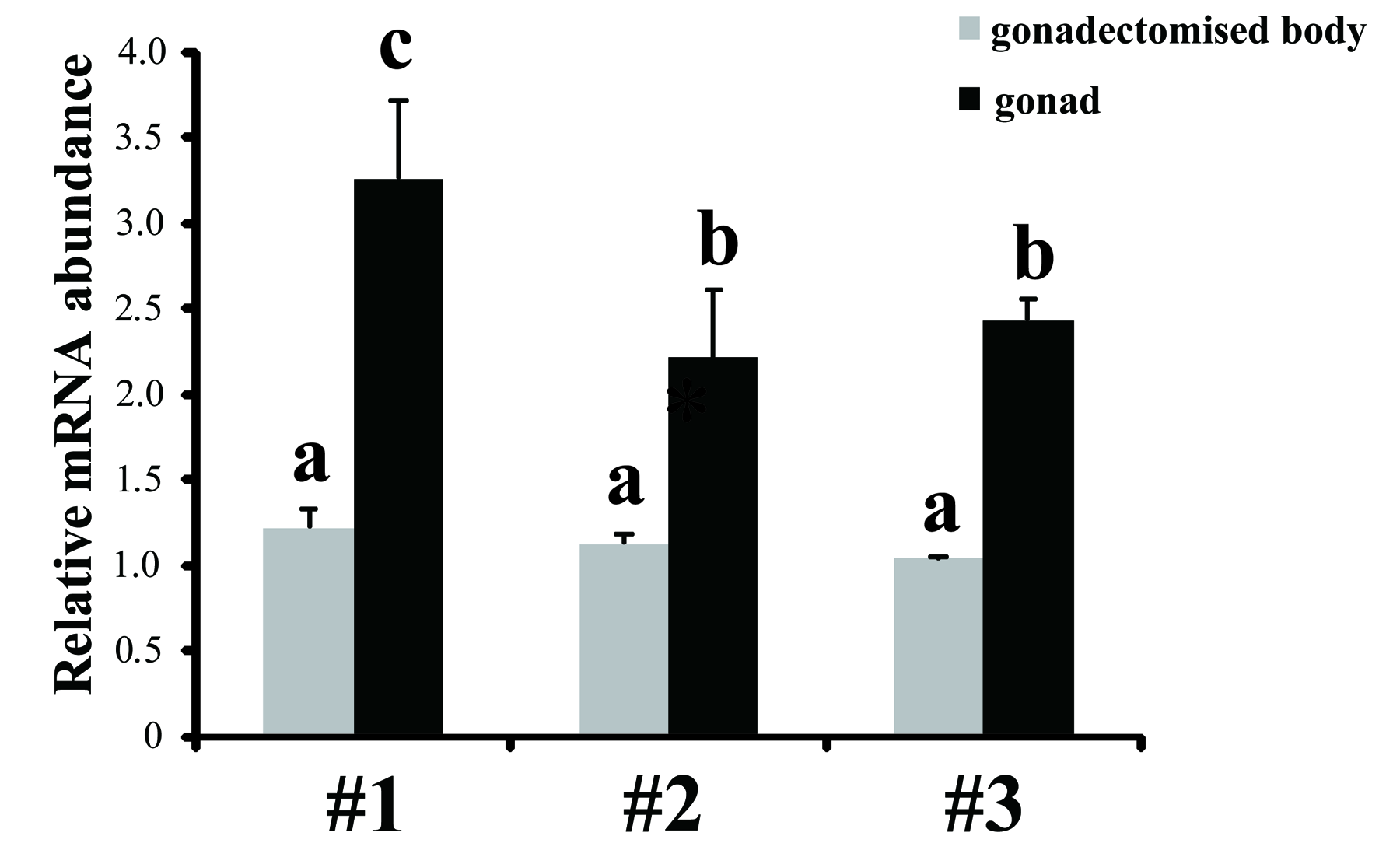

Supplement: S3 Fig — (TIFF) [file pone.0123900.s003.tiff]

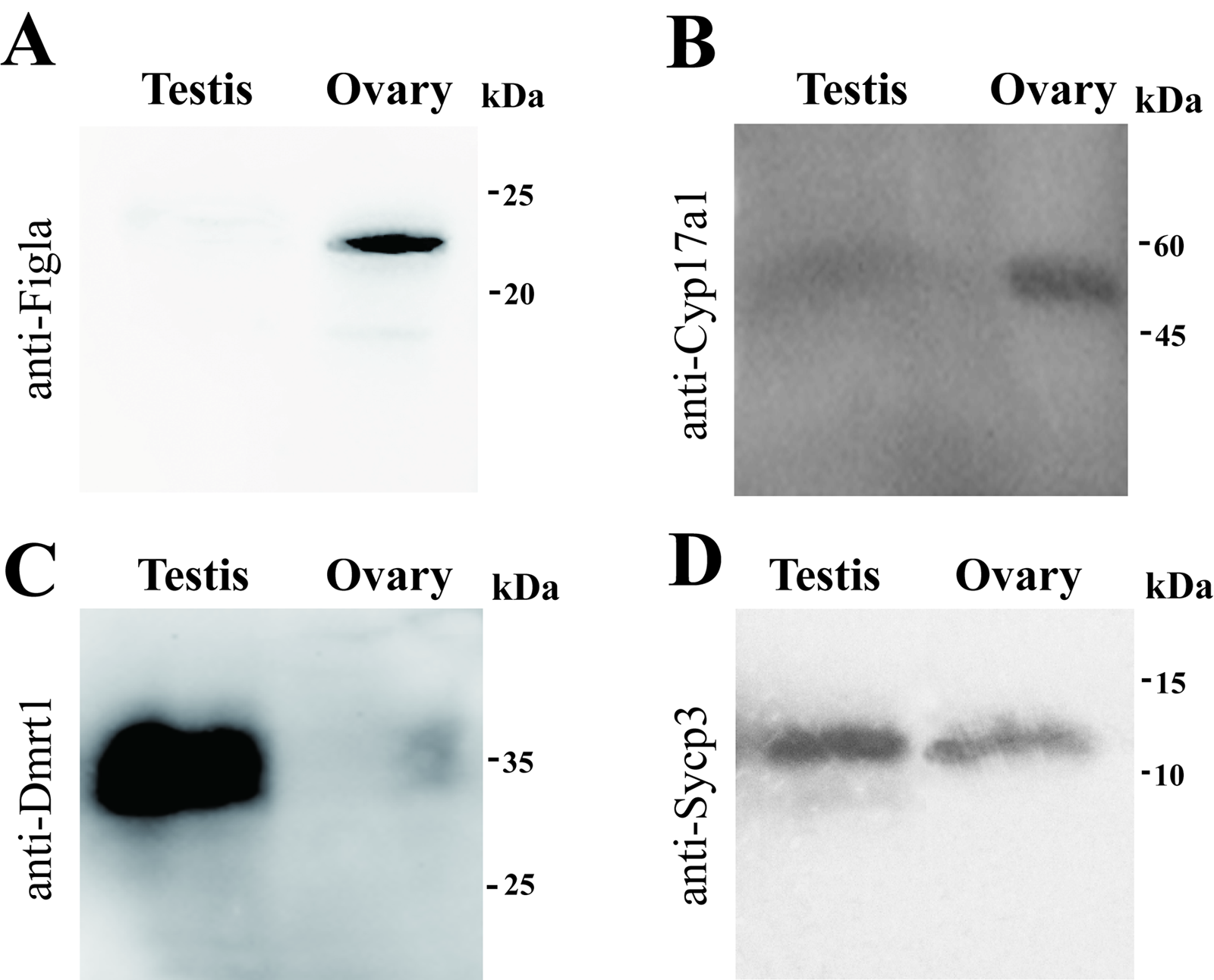

Supplement: S4 Fig — (TIFF) [file pone.0123900.s004.tiff]
