# Supplementary material for: Figla Favors Ovarian Differentiation by Antagonizing Spermatogenesis in a Teleosts, Nile Tilapia (Oreochromis niloticus)
Source: PLoS One. 2015 Apr 20;10(4):e0123900. doi: 10.1371/journal.pone.0123900 (PMC4404364; doi:10.1371/journal.pone.0123900)
Supplement: S1 Table — (DOC) [file pone.0123900.s005.doc]

**S1 Table. Primer sequences used for molecular cloning and PCR amplification.**

| Primers | Sequences (5’ to 3’) | Purpose |
| --- | --- | --- |
| *Figl*a-o-F | ATGAAGGTACCGGAGGAGGAA | Amplification |
| *Figl*a-o-R | CCAGGCTTCTAATAGGCCTAC |  |
| *Figla*-ov-F | GGAATTCCATGAAGGTACCGGAGGAGGAAT | Overexpression |
| *Figla*-ov-R | CCGCTCGAGCGGGCAGGGTTGAGACGTCGCTG |  |
| *Figla*-F | GGAGTTCAGCGAAACTGTGA | Real-time |
| *Figla*-R | AGCGGGAGAACATGGTATT |  |
| *gapdh*-F | AAGCTCATTTCCTGGTAT | Internal control |
| *gapdh*-R | CCTTTGCTGATTTCCTTG |  |
| *eef1a*-F | CAAGTGCGGAGGAATCGA |  |
| *eef1a*-R | CGAACTTCCACAGAGCGATA | . |
| *actb*-F | GGCATCACACCTTCTACAACGA |  |
| *actb*-R | ACGCTCTGTCAGGATCTTCA |  |
| *hrGFP*-F | ATGGTGAGCAAGCAGATCCTG | Fish screening |
| *hrGFP*-R | TTACACCCACTCGTGCAGGCT |  |
| T3 | AATTAACCCTCACTAAAGGG |  |
