# Supplementary material for: Figla Favors Ovarian Differentiation by Antagonizing Spermatogenesis in a Teleosts, Nile Tilapia (Oreochromis niloticus)
Source: PLoS One. 2015 Apr 20;10(4):e0123900. doi: 10.1371/journal.pone.0123900 (PMC4404364; doi:10.1371/journal.pone.0123900)
Supplement: S2 Table — (DOC) [file pone.0123900.s006.doc]

**S2 Table. Integration and abnormality rates of *Figla*-transgene XY tilapia.**

| Gene | Plasmid  concentation(ng/ul） | Survived fish | Integration rate | Integration rate |
| --- | --- | --- | --- | --- |
| *Figla* | 80 | 42 | 47.62%(20/42) | 95%(19/20) |
